# Overview of Cochrane Systematic Reviews on Interventions for Rehabilitation in People with Ischemic Heart Disease: A Mapping Synthesis

**DOI:** 10.3390/jcm13133662

**Published:** 2024-06-23

**Authors:** Matteo Johann Del Furia, Chiara Arienti, Gaia Cattadori, Silvia Di Marco, Carlotte Kiekens

**Affiliations:** 1 Department of Biomedical, Surgical and Dental Sciences, University of Milan, 20122 Milan, Italy; matteojohann.delfuria@gmail.com; 2 Department of Mental and Physical Health and Preventive Medicine, University of Campania Luigi Vanvitelli, 80138 Naples, Italy; 3 IRCCS Istituto Ortopedico Galeazzi, 20157 Milan, Italy; carlotte.kiekens@isico.it; 4 Clinical Epidemiology and Research Center, Department of Biomedical Sciences, Humanitas University, Piave Emanuele, 20090 Milan, Italy; chiara.arienti@hunimed.eu; 5 Department of Clinical Sciences and Community Health, University of Milan, 20122 Milan, Italy; 6 IRCCS MultiMedica, 20138 Milan, Italy; silvia.dimarco@multimedica.it

**Keywords:** myocardial ischemia, rehabilitation, systematic reviews as topic, evidence-based practice

## Abstract

**Objectives:** This overview of Cochrane Systematic Reviews (CSRs) reports on current evidence and its certainty of the effectiveness of interventions for the rehabilitation of people with ischemic heart disease (IHD), included in the World Health Organization Rehabilitation Programme Package of Interventions for Rehabilitation. **Methods:** We included all the CSRs relevant to people with IHD. We used a mapping synthesis to group outcomes and comparisons of included CSRs, indicating the effectiveness of interventions for rehabilitation and the certainty of evidence. **Results:** The evidence map included a total of 13 CSRs. The effect of the interventions varied across comparisons, and the certainty of evidence was inconsistent, ranging from high to very low. We found the best evidence for exercise-based cardiac rehabilitation in the reduction of fatal and non-fatal myocardial infarction and all-cause hospital admission up to 12 months follow-up. Also, combined interventions (work-directed interventions, physical conditioning interventions, and psychological interventions) reduce the days needed for returning to work. **Conclusions:** The current effect and certainty of evidence for several comparisons investigated support the role of exercise-based cardiac rehabilitation in the management of people with IHD, specifically reducing the risk of fatal and non-fatal myocardial infarction and hospitalisation. However, our findings highlight the lack of high-certainty evidence about hard endpoints, particularly total mortality. Future research should prioritise these primary endpoints to enhance the credibility of cardiac rehabilitation.

## 1. Introduction

In 2017, the World Health Organization (WHO) launched the “Rehabilitation 2030: a call for action” initiative to strengthen rehabilitation in health systems and integrate it into all levels of health care [1]. Within this initiative, the WHO Rehabilitation Programme developed a Package of Interventions for Rehabilitation (WHO PIR) that was launched at the third Rehabilitation 2030 meeting held in Geneva in July 2023 [2]. This package responds to the global need for rehabilitation services, which should be integrated into primary health care to reach more people in need. The development of the PIR was composed of six consecutive phases. In the first phase, the WHO Rehabilitation Programme Advisory Board selected 20 health conditions in the summer of 2018 based on the disability statistics of the Global Burden of Disease (GBD) Study 2016 [3] and expert opinion, according to two criteria: (1) to be amenable to rehabilitation and (2) to cover different disease areas (e.g., musculoskeletal, cardiovascular, nervous system). In addition, the level of disability associated with these health conditions and their prevalence estimates were considered [4]. Phase 2, called “Best-Evidence for Rehabilitation (be4rehab)”, aimed to identify the best quality evidence concerning the effectiveness of interventions for rehabilitation for 20 key health conditions, including ischemic heart disease.

Ischemic heart disease (IHD), also called coronary heart disease or coronary artery disease, is the single most common cause of death globally, with over 9 million deaths per year worldwide [5]. According to the American Heart Association, the annual incidence of new coronary events is approximately 720,000 cases per year, and the current prevalence of IHD is approximately 18.2 million cases in the United States alone [6]. The morbidity has social and economic implications. According to the study of Leal et al. 2006 [7], it is estimated that for the European Union, the total costs were around EUR 45 billion in 2003, broken down as follows: 51% incurred in health care, 34% in productivity losses, and 15% in informal care. In addition, anxiety and depression commonly occur after IHD, affecting the quality of life (QoL) and return to work of the persons concerned.

Rehabilitation plays an important role in the overall management of people with IHD. Exercise training is commonly recommended in the management of people with IHD as a core element of a Cardiac Rehabilitation Program [8,9,10].

Cochrane Reviews are the reference standard among systematic reviews for their methodological rigour and quality [11]. The evidence gathered from these reviews is the strongest available and was consequently considered by the WHO as highly relevant for PIR development. Due to the methodological rigour and quality of the CSRs, this paper aims to describe the research performed and the Cochrane evidence found on interventions for the rehabilitation of people with IHD. The results provide an overall description of the available evidence in the field through a specific methodology still not widely used in medicine—an evidence map [12].

## 2. Materials and Methods

The methods for developing the content of the WHO PIR have been established and published collaboratively by the WHO Rehabilitation Programme and Cochrane Rehabilitation under the guidance of the WHO’s guideline review committee [4]. A first search of the Cochrane Systematic Reviews (CSRs) relevant to the WHO PIR for IHD was carried out in August 2019 on the Cochrane Rehabilitation database of tagged CSRs [13], according to the Preferred Reporting Items for Systematic Reviews and Meta-analyses (PRISMA) Guidelines [14]. The data were extracted and communicated to the WHO for the development of the PIR. In a second instance, to overcome the limitations of the rapid overview of reviews requested by the WHO, we performed a search on the full Cochrane Library on 11 December 2023, according to the protocol submitted on OSF registers (10.17605/OSF.IO/TPABF). We included only CSRs that examined interventions for rehabilitation in people with IHD. Studies focusing on interventions that are not related to rehabilitation were excluded.

### 2.1. Search Strategy

The first search was performed according to the methodology developed by the WHO and Cochrane Rehabilitation for the WHO PIR [4,15]. In the second search, we identified studies from searches of the Cochrane Central Register of Controlled Trials (CENTRAL), published in the Cochrane Library. Detailed search strategies have been developed by an author with experience in bibliographic searches (MJDF), using the terms “myocardial ischemia” and “myocardial revascularisation”. The database was searched on 11 December 2023. The full search strategy is shown in Table 1.

### 2.2. Study Selection and Data Extraction

Two independent authors (CK, GC) screened articles for eligibility at the title/abstract stage and the full-text stage. They selected the CSRs relevant to rehabilitation, considering all reviews on interventions provided or prescribed by rehabilitation professionals. In case of disagreement, a third author was involved (CA). One author (MJDF) performed data extraction, and two authors (CK, GC) verified the accuracy of the process. We extracted all rehabilitation-relevant interventions from the table of findings published in each CSR and collated them into an Excel file datasheet the PICO (population, intervention, comparison, outcome) information, as follows: type of outcomes and outcome measures, number of included studies, sample size, population, intervention, comparison, effect (in favour of intervention, in favour of control, no effect), and the certainty of evidence judgement for each comparison and outcome.

### 2.3. Assessment of Methodological Quality of Included Reviews

To assess the methodological quality of the included reviews, we used the 16-item AMSTAR 2 (A MeaSurement Tool to Assess systematic Reviews), published in 2007 and updated in 2017 [16]. The tool consists of binary (yes or no) questions and does not produce an overall score. The overall rating is determined based on weaknesses identified in seven critical domains. To evaluate the weaknesses, two independent authors (MJDF, CK) critically appraised the included reviews. If there was a disagreement, a third author was consulted to reach a consensus (CA).

### 2.4. Certainty of Evidence Appraisal

We extracted the Grading of Recommendations Assessment, Development, and Evaluation (GRADE) judgement of each CSR. When CSRs lacked this information, two authors independently applied the standard GRADE approach to evaluate the quality of evidence for the primary outcomes [17,18]. This post hoc GRADE process comprised the retrieval of the original primary studies included in each CSR and tabulation of the judgments in Summary of Findings tables using the GRADEPro software.

### 2.5. Summarising Evidence within a Map

After the data extraction, we summarised the results into an evidence map, a specific methodology used to identify the literature within a research field to provide a comprehensive view of what is known and where evidence gaps exist [12,19]. An Excel sheet was used to map the evidence, grouping outcomes, and comparison of included CSRs indicating the effect (no, in favour of intervention, in favour of control) and the quality of evidence (very low, low, moderate, and high).

## 3. Results

**Figure 1:**
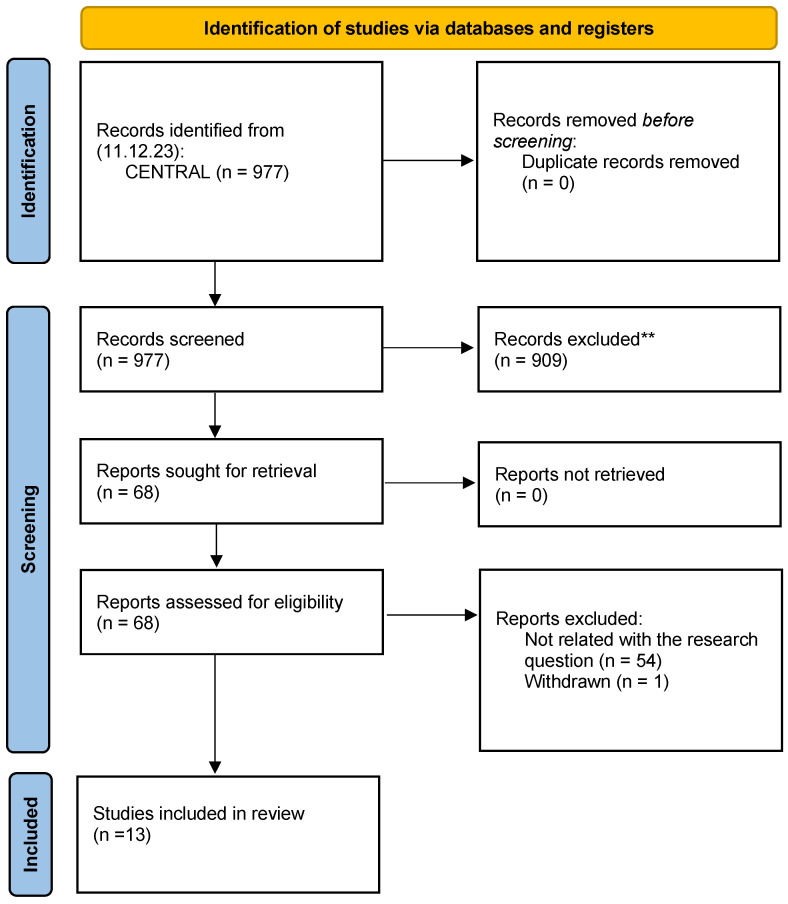
PRISMA flow diagram of the study. ** If automation tools were used, indicate how many records were excluded by a human and how many were excluded by automation tools.

**Figure 2:**
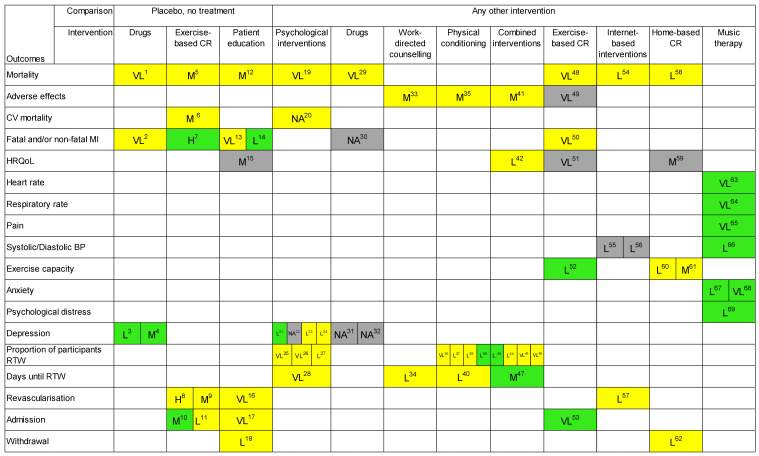
Evidence map for interventions for persons with ischemic heart disease. Map colours: green: favour intervention; yellow: no effect; grey: not estimable; Abbreviations for certainty of evidence: VL: very low; L: low; M: moderate; H: high; NA: not available. Other abbreviations: ischemic heart disease: IHD; myocardial Infarction: MI; Coronary Artery Bypass Grafting: CABG; percutaneous coronary intervention: PCI; health-related quality of life: HRQoL; cardiac rehabilitation: CR; cardiovascular: CV; returning to work: RTW; blood pressure: BP. Outcomes (comparison) legend: 1: all-cause mortality (non-pharmacological interventions); 2: myocardial infarction (placebo); 3: depression symptoms: objective and self-reported measures of depression symptoms—short term (placebo); 4: depression remission: Hamilton rating Scale for Depression—short term (placebo); 5: all-cause mortality (‘no exercise’ control); 6: cardiovascular mortality (‘no exercise’ control); 7: fatal and/or non-fatal MI (‘no exercise’ control); 8: CABG (‘no exercise’ control); 9: PCI (‘no exercise’ control); 10: all-cause hospital admission (‘no exercise’ control); 11: cardiovascular hospital admission (‘no exercise’ control); 12: total mortality (no patient education); 13: fatal and/or non-fatal MI at the end of the follow-up period (no patient education); 14: other fatal and/or non-fatal cardiovascular events (no patient education); 15: HRQoL (no patient education); 16: total revascularisations, including CABG and PCI (no patient education); 17: hospitalisations—cardiac-related—at end of follow-up period (no patient education); 18: all-cause withdrawal at follow-up (no patient education); 19: all-cause mortality—short term (usual care); 20: cardiovascular mortality—long term (usual care); 21: depression symptoms—short term (usual care); 22: depression remission—short term (usual care); 23: depression symptoms—short term (end of treatment) (other psychological treatment); 24: depression remission—short term (end of treatment) (other psychological treatment); 25: proportion of participants returning to work in the short term—up to 6 months (lesser forms of cardiovascular rehabilitation); 26: proportion of participants returning to work in the medium term—from 6 months to 1 year (lesser form of cardiovascular rehabilitation); 27: proportion of participants at work in the long term—from >1 to <5 years (lesser forms of cardiovascular rehabilitation); 28: days until return to work (lesser forms of cardiovascular rehabilitation); 29: all-cause mortality—short term, end of treatment (other pharmacological intervention); 30: myocardial infarction—short term, end of treatment (other pharmacological intervention); 31: depression symptoms—short term, end of treatment (other pharmacological treatment); 32: depression remission—short term, end of treatment (other pharmacological intervention); 33: adverse effects: cardiac deaths (usual care); 34: days until return to work (usual care); 35: adverse effects: cardiac deaths (usual care); 36: proportion of participants returning to work in the short term—up to 6 months (usual care); 37: proportion of participants returning to work in the medium term—from 6 months to 1 year (usual care); 38: proportion of participants at work in the long term—from >1 to <5 years (usual care); 39: proportion of participants at work in the extended long term ≥ 5 years (usual care); 40: days until return to work (usual care); 41: adverse effects: reinfarctions (usual care); 42: health-related quality of life (usual care); 43: proportion of participants returning to work in the short term—up to 6 months (usual care); 44: proportion of participants returning to work in the medium term—from 6 months to 1 year (usual care); 45: proportion of participants at work in the long term—from >1 to <5 years (usual care); 46: proportion of participants at work in the extended long term ≥ 5 years (usual care); 47: days until return to work (usual care); 48: all-cause mortality (standard medical care but any structured training or advice on structured exercise training); 49: adverse events (standard medical care but without any structured training or advice on structured exercise training); 50: acute MI (standard care, such as drug therapy, but without any form of structured exercise training or advice); 51: HRQoL (standard care without any form of structured exercise training or advice); 52: exercise capacity (standard care, such as drug therapy, but without any form of structured exercise training or advice); 53: cardiovascular-related hospital admissions (standard care, such as drug therapy, but without any form of structured exercise training or advice); 54: total mortality (usual care or no care); 55: systolic blood pressure (usual care or no care); 56: diastolic blood pressure (usual care or no care); 57: revascularisation (usual care or no care); 58: total mortality (centre-based cardiac rehabilitation); 59: HRQoL (centre-based cardiac rehabilitation); 60. exercise capacity ≤ 12 months (centre-based cardiac rehabilitation); 61: exercise capacity > 12 months (centre-based cardiac rehabilitation); 62: withdrawal from the intervention group (centre-based cardiac rehabilitation); 63: heart rate (standard care); 64: respiratory rate (standard care); 65: pain (standard care); 66: systolic blood pressure (standard care); 67: state anxiety in MI patients—STAI (standard care); 68: anxiety—NRS, VAS, HADS, STAI (standard care); 69: psychological distress (standard care).

Among the 977 CSRs, 13 met the inclusion criteria set by the WHO (see Figure 1). Table 2 shows the characteristics of the included CSRs. The results of the AMSTAR 2 assessment indicated a high methodological quality of all the CSRs (see Table S1). Nine reviews reported the certainty of evidence using the GRADE approach [20,21,22,23,24,25,26,27,28], while the other four did not report GRADE judgement [29,30,31,32]. The included CSRs investigated interventions for the treatment of the rehabilitation needs in patients with IHD on 28 outcomes analysed within 13 comparisons in inpatient and outpatient settings. The findings were grouped by outcome and arbitrarily classified according to the most appropriate International Classification of Functioning Disability and Health (ICF) category [33]. The results were reported into an evidence map to ensure easy readability of the information (Figure 2).

### 3.1. All-Cause Mortality, Cardiovascular Mortality, Fatal and/or Non-Fatal Myocardial Infarction [Heart Function: b410]

#### 3.1.1. High- and Moderate-Certainty Evidence

Exercise-based CR, compared to no exercise treatment, results in a large reduction in fatal and/or non-fatal myocardial infarction up to 12 months follow-up (high certainty), but it has no cardiovascular effect, up to 12 months’ follow-up, or total mortality effect (moderate certainty) [23]. Patient education alone, compared with no treatment, probably gives no reduction in total mortality (moderate certainty) [20].

#### 3.1.2. Low- and Very-Low-Certainty Evidence

Internet-based interventions, compared to usual care or no intervention, may have no effect on mortality (low certainty). Home-based treatment, compared to centre-based CR, may have no effect on mortality up to 12 months (low certainty) [26]. It is uncertain whether patient education reduces fatal and/or non-fatal MI compared to no exercise treatment (very low certainty), while it may reduce other fatal or non-fatal cardiovascular events (low certainty) [20]. Similarly, psychological treatment probably has no effect on total mortality when compared to usual care (very low certainty) [28]. Finally, there are uncertain effects of exercise-based CR on acute MI [28].

### 3.2. Hospitalisation with or without Revascularization Procedures (Coronary Artery Bypass Graft [CABG], Percutaneous Coronary Intervention [PCI]) [No ICF Classification]

#### 3.2.1. High- and Moderate-Certainty Evidence

Exercise-based CR, compared with no exercise treatment, probably reduces all-cause hospital admissions up to 12 months follow-up (moderate certainty), while it has no effect on CABG revascularisation (high certainty) and probably no effect on the risk of PCI revascularisation (moderate certainty) up to 12 months follow-up [23].

#### 3.2.2. Low- and Very-Low-Certainty Evidence

The effect of exercise-based CR compared to no treatment and standard medical care on cardiovascular hospitalisation remains uncertain (low certainty) [23], (very low certainty) [25]. Internet-based interventions, compared to usual care or no care, may have no effect on revascularisation events (low certainty) [22]. Home-based treatment, compared to centre-based CR, may reduce withdrawal from the intervention groups up to 72 months follow-up (low certainty) [26]. Patient education, compared to no treatment, may have no effect on treatment withdrawal of participants (low certainty), while it is uncertain whether it reduces revascularisation events and hospital admissions (very low certainty) [20].

### 3.3. Clinical Outcomes (Health-Related Quality of Life [No ICF Classification], Exercise Capacity [Exercise Tolerance Functions: b455], Heart Rate [Heart Rate: b4100], Respiratory Rate [Respiratory Rate: b4400], Pain [Sensation of Pain: b280], Systolic Blood Pressure [Blood Pressure Function: b420], Diastolic Blood Pressure [Blood Pressure Function: b420])

#### 3.3.1. High- and Moderate-Certainty Evidence

It is uncertain whether home-based CR, compared to centre-based CR, has an effect on health-related QoL up to 24 months follow-up (not estimable; moderate certainty) [26]. Similarly, the effect of patient education on health-related QoL compared to no intervention is uncertain (not estimable, moderate certainty) [20]. There is probably no difference between home-based CR and centre-based CR in exercise capacity > 12 months (moderate certainty) [26].

#### 3.3.2. Low- and Very-Low-Certainty Evidence

Exercise-based CR, compared to standard medical care (such as drug therapy, health education, behavioural or psychological interventions, or surgery, but without any structured exercise training or advice on structured exercise training), may improve exercise capacity up to 12 months follow-up (low certainty), while the effect on health-related QoL is uncertain (not estimable, very low certainty) [25]. Combined interventions (work-directed interventions, physical conditioning interventions, and psychological interventions) may have no effect on health-related QoL compared to usual care (low certainty) [24]. It is uncertain whether internet-based interventions, compared to usual care or no care, have an effect on systolic and diastolic blood pressure (not estimable, low-certainty evidence) [22]. Home-based CR may have no effect on exercise capacity ≤ 12 months, compared to centre-based treatment (low-certainty evidence) [22]. It is uncertain whether music therapy with standard care, compared to standard care only, may have an effect on heart rate, respiratory rate, pain, and systolic blood pressure (very-low-certainty evidence) [21].

### 3.4. Psychological Outcomes (Psychological Distress, Anxiety, Depression Symptoms, Depression Remission) [Emotional Functions: b152]

#### Low- and Very-Low-Certainty Evidence

Psychological treatment, including cognitive behavioural therapy, psychodynamic psychotherapy, interpersonal therapy (IPT), non-directive or supportive therapy, and counselling, compared with usual care, may have an effect on depression symptoms, while no difference was found in depression remission (low-certainty evidence), with potentially no effect. It is uncertain whether music therapy may have an effect on psychological distress and anxiety compared to standard care (low-certainty evidence) [21,28].

### 3.5. Return to Work (Proportion of Participants Returning to Work, Days Until Return to Work) [Remunerative Employment d850; Non-Remunerative Employment d855]

#### 3.5.1. High- and Moderate-Certainty Evidence

Combined interventions (work-directed interventions, physical conditioning interventions, and psychological interventions), compared to usual care, probably show a reduction in the days needed to return to work (moderate certainty) [24]. Finally, combined interventions described as work-directed interventions, person-directed interventions as psychological interventions, and physical conditioning interventions likely result in little to no difference in adverse events, and physical conditioning interventions, compared to usual care, probably do not increase adverse events (such as cardiac deaths or reinfarction) (moderate certainty) if combined with psychological interventions [24].

#### 3.5.2. Low- and Very-Low-Certainty Evidence

Psychological interventions, compared to usual care, may not increase the proportion of participants returning to work in the long term (>1 to <5 years) (low certainty), while it is uncertain whether psychological interventions may increase the proportion of participants returning to work in the short (up to 6 months) and medium term (6 months–1 year) or whether it may lower the days needed to return to work (very low certainty). Work-directed counselling compared to usual care may not reduce the days needed to return to work (low certainty). Physical conditioning interventions, compared to usual care, may increase the proportion of participants returning to work in the very long term (≥5 years) (low certainty evidence), without effects in the medium (6 months–1 year) and long term (>1 to <5 years), and on the days needed to return to work (low-certainty evidence). It is uncertain whether physical conditioning interventions may reduce the proportion of participants returning to work in the short term (up to 6 months) (very-low-certainty evidence). Combined interventions, defined as all work-directed interventions, exercise-based CR, and psychological interventions or any combination, compared to usual care, may increase the proportion of participants returning to work in the short term (up to 6 months) (low-certainty evidence) without raising the proportion of participants returning to work in the medium term (6 months–1 year) (low-certainty evidence). It is uncertain whether combined interventions may increase the proportion of participants returning to work in the long term (very-low-certainty evidence) [24].

### 3.6. Pharmacological Therapy Optimisation

The certainty of evidence of the effects of pharmacological treatment is summarised in Figure 2.

## 4. Discussion

In July 2023, the WHO launched the Package of Interventions for Rehabilitation (WHO PIR), which aimed to identify the best quality evidence concerning the effectiveness of interventions for rehabilitation of cardiopulmonary conditions [2]. The PIR individualised the interventions for rehabilitation as crucial to achieve, restore, and maintain optimal levels of functioning and to prevent additional cardiac events. In this paper, we mapped the Cochrane evidence on the effectiveness of rehabilitation interventions for people with IHD, addressing all core components included in the PIR: exercise training, patient education, psychological intervention, and pharmacological optimisation, including different modes of delivery such as internet-based, home-based, or centre-based. Our data show that exercise-based CR, compared to no exercise treatment, provides a large reduction in fatal and non-fatal myocardial infarction up to 12 months of follow-up and probably reduces all-cause hospital admissions up to 12 months of follow-up, while combined interventions (work-directed interventions, physical conditioning interventions, and psychological interventions) provide a reduction in the days needed for returning to work. These findings align with those of a recently published network meta-analysis, which indicates a decrease in the risk of non-fatal myocardial infarction [34].

However, our overview shows that CR has no effect on both total and cardiovascular mortality, considering exercise-based CR, patient education or psychological treatment alone. Moreover, exercise-based CR shows no effect on CABG revascularisation and risk of PCI revascularisation up to 12 months. Patient education alone provides uncertain effects on health-related QoL and the reduction of fatal and non-fatal MI compared to no treatment, while it may reduce other fatal or non-fatal cardiovascular events. Finally, when evaluating CR settings, home-based CR, compared to centre-based CR, gives uncertain effects on the health-related QoLup to 24 months follow-up, and very-low-certainty evidence exists on the effect on mortality up to 12 months. Similarly, low-certainty evidence exists about internet-based interventions, compared to usual care or no intervention.

Our data support recent international guidelines and meta-analyses. The 2023 AHA/ACC/ACCO/ASPC/NLA/PCNA Guidelines for the management of patients with chronic coronary disease [10] recommend all patients with chronic coronary disease to be referred to a CR program to improve outcomes. A CR programme is generally considered a multidisciplinary intervention with exercise training as a pivotal part. General physical activity recommendations are reported, including a combination of regular aerobic physical activity and resistance exercise throughout the week, without any data about outcomes specifically after exercise-based CR. The 2023 ESC Guidelines for the management of acute coronary syndromes [9] in the “Long treatment” Section recommend that all acute coronary syndrome patients participate in a comprehensive CR programme to reduce CV hospitalisations, MI (in line with our data) and CV mortality. Regarding the outcome of mortality, it is specified “in some studies”, suggesting the paucity of data supporting this last hard endpoint. Our data show with moderate certainty that exercise-based CR versus non-treatment or other treatment has no effect on cardiovascular and total mortality. Evaluating CR settings, both European and American guidelines report telerehabilitation as an alternative for patients who cannot attend facility-based CR, being equivalent to traditional CR in terms of achieving functional improvement, managing risk factors and increasing well-being. In line with our data, few data are available about the effect of telerehabilitation on recurrent events and only one meta-analysis showed no significant difference between mortality following telehealth intervention and centre-based supervised CR.

Finally, a recently published meta-analysis [35] focusing on exercise-based CR for coronary heart disease (CHD) reported data about 85 RCTs involving 23,430 patients with a median 12-month follow-up. Perfectly in line with our findings, exercise-based CR was associated with significant risk reductions in hospitalisations and MI without significant impact on all-cause mortality and the need for coronary artery bypass graft or percutaneous coronary intervention. The take-home message of the important benefit of exercise-based CR to reduce risk of cardiovascular mortality, apparently contrary to our data, is not detected at short-term follow-up (6–12 months), and the certainty/quality of data is not reported.

Our findings reinforce the current national and international clinical directives emphasising that effective CR for those with CHD should be comprehensive and include educational interventions in conjunction with exercise and psychological therapy [36]. The current landscape, as highlighted in the articles by Vilela et al. and Beatty et al. [37,38], underscores the evolving role of CR in managing CHD. Prospects suggest a shift towards personalised, patient-centred approaches that integrate technology, behavioural interventions, and novel exercise modalities tailored to individual needs. Additionally, there is a growing emphasis on addressing research gaps, such as optimising modes of delivery, understanding the impact of psychosocial factors on rehabilitation outcomes, and exploring innovative strategies to improve adherence to long-term programmes. Advancements in wearable technology, telemedicine, and data analytics are expected to play a pivotal role in monitoring and optimising patient progress outside clinical settings, thereby enhancing the overall effectiveness of cardiac rehabilitation. Collaboration between multidisciplinary teams, including cardiologists, rehabilitation physicians, exercise physiologists, physiotherapists, psychologists, dietitians, and technology experts, will likely drive the development of comprehensive and adaptive rehabilitation programmes. Ultimately, the future of CR research holds promise in redefining and optimising the standard of care for individuals with CHD, paving the way for more effective, accessible, and personalised rehabilitation approaches.

### Strengths and Limitations

Our findings result from selecting CSRs according to the methods framed by the WHO Rehabilitation Programme and Cochrane Rehabilitation. We included CSRs exclusively as they represent the gold standard among systematic reviews due to their high-quality methodology; however, this could limit the generalizability of the findings and investigated interventions. Nevertheless, the uniformity of the Cochrane methodology gives coherence to the overview and is currently suggested by the WHO. Not providing a full evidence map that should start from an “a priori” grid developed according to a specific methodology, including all the possible outcomes and interventions, is a limitation. Moreover, according to the reported interventions and outcomes, we provided only the GRADE evidence of the current CSRs. Although beyond the scope of this paper, the authors acknowledge that this did not allow them to identify the evidence gaps fully. Given the heterogeneity in the interventions for rehabilitation observed in the included studies, further research to refine the optimal cardiac rehabilitation approach for individuals with IHD is needed. Also, as discussed in the CSRs, well-designed and adequately reported RCTs more representative of usual clinical practise are still needed. Additionally, to ensure improved comparability between trials, it will be important to define a core set of outcomes to be included in all rehabilitation studies for IHD patients. Finally, with a view towards personalised treatment, it will be crucial to examine the effectiveness of various rehabilitation interventions in IHD patients, taking into account diverse clinical phenotypes, disease severity, and comorbidities.

## 5. Conclusions

In this paper we provided the most reliable evidence on rehabilitation interventions for persons with IHD, which is included in the WHO PIR. The current effect and certainty of evidence for several comparisons investigated in this population support the role of exercise-based cardiac rehabilitation in the management of people with IHD. However, our data highlight the lack of high-certainty evidence about hard endpoints, in particular CV and all-cause mortality, which must be the main priority of future research to improve the credibility of CR, which should be much more integrated into primary health care and the overall management of people with IHD. Moreover, many different aspects, such as modes of delivery, the impact of psychosocial factors on rehabilitation outcomes, innovative strategies to improve adherence to long-term programmes, wearable technology, and telemedicine, should be included in future studies.

## Figures and Tables

**Table 1:** Search strategy.

**Database**	**Search Strategy**
CENTRAL (via Cochrane Library)	“ Myocardial Ischemia”[Mesh] OR “Myocardial Revascularization “ [Mesh] (myocard* NEAR/1 isch*mi*):ti,ab,kw (isch*mi* NEAR/1 heart):ti,ab,kw angina:ti,ab,kw (angina NEXT pectoris):ti,ab,kw (myocard* NEAR/1 infarct*):ti,ab,kw (heart NEAR/1 infarct*):ti,ab,kw (myocard* NEAR/1 revascularization):ti,ab,kw coronary:ti,ab,kw (coronary NEAR/1 disease*):ti,ab,kw myocard*:ti,ab,kw cardiac*:ti,ab,kw #1 OR #2 OR #3 OR #4 OR #5 OR #6 OR #7 OR #8 OR #9 OR #10 OR #11 OR #12—in Cochrane Reviews

**Table 2:** Characteristics of the Cochrane Systematic Reviews included.

CSRs Authors	Title	Total N° of Included Studies (N° Participants)	Population	Setting	Intervention	Control	Outcome	Outcome Measurements	N° Studies (N° Participants)	Effect	GRADE
Anderson, 2017 [20]	Patient education in the management of CHD	22 (76,864)	People with CHD	Mixed (Centre, home-based)	Patient education	No education	Total mortality	NR	13 (10,075)	No effect	MODERATE
Anderson, 2017 [20]	Patient education in the management of CHD	22 (76,864)	People with CHD	Mixed (Centre, home-based)	Patient education	No education	Fatal and/or non-fatal MI	NR	2 (209)	No effect	VERY LOW
Anderson, 2017 [20]	Patient education in the management of CHD	22 (76,864)	People with CHD	Mixed (Centre, home-based)	Patient education	No education	Other fatal and/or non-fatal cardiovascular events	NR	2 (310)	Favour intervention	LOW
Anderson, 2017 [20]	Patient education in the management of CHD	22 (76,864)	People with CHD	Mixed (Centre, home-based)	Patient education	No education	Total revascularisation (including CABG and PCI)	NR	3 (456)	No effect	LOW
Anderson, 2017 [20]	Patient education in the management of CHD	22 (76,864)	People with CHD	Mixed (Centre, home-based)	Patient education	No education	Hospitalisation (cardiac-related)	NR	5 (14,849)	No effect	VERY LOW
Anderson, 2017 [20]	Patient education in the management of CHD	22 (76,864)	People with CHD	Mixed (Centre, home-based)	Patient education	No education	All-cause withdrawal	NR	17 (10,972)	No effect	LOW
Anderson, 2017 [20]	Patient education in the management of CHD	22 (76,864)	People with CHD	Mixed (Centre, home-based)	Patient education	No education	HRQoL	Various HRQoL measures	13 (4393)	Not pooled	MODERATE
Barth, 2015 [29]	Psychosocial interventions for smoking cessation in patients with CHD	40	Patients with CHD	NR	Psychosocial intervention	UC		NR	Not measured	NR	Not estimable
Bradt, 2013 [21]	Music for stress and anxiety reduction in CHD patients	26 (1369)	People with CHD	Mixed (inpatients, outpatients)	Music therapy	Standard care	Psychological Distress	Profile of Mood States (POMS)	5 (228)	Favour intervention	LOW
Bradt, 2013 [21]	Music for stress and anxiety reduction in CHD patients	26 (1369)	People with CHD	Mixed (inpatients, outpatients)	Music therapy	Standard care	Anxiety (all measures)	NRS, VAS, Hospital Anxiety and Depression Scale (HADS), STAI	10 (353)	Favour intervention	VERY LOW
Bradt, 2013 [21]	Music for stress and anxiety reduction in CHD patients	26 (1369)	People with CHD	Mixed (inpatients, outpatients)	Music therapy	Standard care	State anxiety (MI patients)	STAI	6 (243)	Favour intervention	LOW
Bradt, 2013 [21]	Music for stress and anxiety reduction in CHD patients	26 (1369)	People with CHD	Mixed (inpatients, outpatients)	Music therapy	Standard care	Heart rate	bpm	13 (828)	Favour intervention	VERY LOW
Bradt, 2013 [21]	Music for stress and anxiety reduction in CHD patients	26 (1369)	People with CHD	Mixed (inpatients, outpatients)	Music therapy	Standard care	Respiratory rate	Breaths per minute	7 (442)	Favour intervention	VERY LOW
Bradt, 2013 [21]	Music for stress and anxiety reduction in CHD patients	26 (1369)	People with CHD	Mixed (inpatients, outpatients)	Music therapy	Standard care	Systolic blood pressure	NR	11 (775)	Favour intervention	LOW
Bradt, 2013 [21]	Music for stress and anxiety reduction in CHD patients	26 (1369)	People with CHD	Mixed (inpatients, outpatients)	Music therapy	Standard care	Pain	VAS, NRS	8 (562)	Favour intervention	VERY LOW
Devi, 2015 [22]	Internet-based interventions for the secondary prevention of CHD	18 (1392)	Patients with CHD	Health care settings	Internet-based interventions	UC or no care	Total mortality	NR	6 (895)	No effect	LOW
Devi, 2015 [22]	Internet-based interventions for the secondary prevention of CHD	18 (1392)	Patients with CHD	Health care settings	Internet-based interventions	UC or no care	Revascularisation	NR	6 (895)	No effect	LOW
Devi, 2015 [22]	Internet-based interventions for the secondary prevention of CHD	18 (1392)	Patients with CHD	Health care settings	Internet-based interventions	UC or no care	Systolic blood pressure	NR	5 (623)	Not pooled	LOW
Devi, 2015 [22]	Internet-based interventions for the secondary prevention of CHD	18 (1392)	Patients with CHD	Health care settings	Internet-based interventions	UC or no care	Diastolic blood pressure	NR	5 (622)	Not pooled	LOW
Dibben, 2021 [23]	Exercise-based cardiac rehabilitation for CHD	22 (7795)	People with CHD	Hospital-based, community-based and home-based settings	Exercise-based CR	No exercise control	All-cause mortality	mortality records	25 (8823)	No effect	MODERATE
Dibben, 2021 [23]	Exercise-based CR for CHD	22 (7795)	People with CHD	Hospital-based, community-based and home-based settings	Exercise-based CR	No exercise control	Cardiovascular mortality	mortality records	1 (16)	No effect	MODERATE
Dibben, 2021 [23]	Exercise-based CR for CHD	22 (7795)	People with CHD	Hospital-based, community-based and home-based settings	Exercise-based CR	No exercise control	Fatal and/or non-fatal MI	NR	22 (7423)	Favour intervention	HIGH
Dibben, 2021 [23]	Exercise-based CR for CHD	22 (7795)	People with CHD	Hospital-based, community-based and home-based settings	Exercise-based CR	No exercise control	Revascularisation-CABG	NR	20 (4473)	No effect	HIGH
Dibben, 2021 [23]	Exercise-based CR for CHD	22 (7795)	People with CHD	Hospital-based, community-based and home-based settings	Exercise-based CR	No exercise control	Revascularisation-PCI	NR	13 (3465)	No effect	MODERATE
Dibben, 2021 [23]	Exercise-based CR for CHD	22 (7795)	People with CHD	Hospital-based, community-based and home-based settings	Exercise-based CR	No exercise control	All-cause hospital admissions	NR	14 (2030)	Favour intervention	MODERATE
Dibben, 2021 [23]	Exercise-based CR for CHD	22 (7795)	People with CHD	Hospital-based, community-based and home-based settings	Exercise-based CR	No exercise control	Cardiovascular hospital admission	NR	6 (1087)	No effect	LOW
Hegewald, 2019 [24]	Interventions to support return to work for people with CHD	39 (8857)	People with CHD	Inpatients, outpatients	Psychological interventions (including health education)	UC	Proportion of participants returning to work in the short term (up to 6 months)	Event data (return-to-work rates, disability pension rates) or time-to-event data (time span between reporting sick and resumption of work, number of days on sick leave during the follow-up period)	6 (375)	No effect	VERY LOW
Hegewald, 2019 [24]	Interventions to support return to work for people with CHD	39 (8857)	People with CHD	Inpatients, outpatients	Physical conditioning interventions	UC	Proportion of participants returning to work in the short term (up to 6 months)	Event data (return-to-work rates, disability pension rates) or time-to-event data (time span between reporting sick and resumption of work, number of days on sick leave during the follow-up period)	4 (460)	No effect	VERY LOW
Hegewald, 2019 [24]	Interventions to support return to work for people with CHD	39 (8857)	People with CHD	Inpatients, outpatients	Combined interventions	UC	Proportion of participants returning to work in the short term (up to 6 months)	Event data (return-to-work rates, disability pension rates) or time-to-event data (time span between reporting sick and resumption of work, number of days on sick leave during the follow-up period)	4 (395)	Favour intervention	LOW
Hegewald, 2019 [24]	Interventions to support return to work for people with CHD	39 (8857)	People with CHD	Inpatients, outpatients	Psychological interventions (including health education)	UC	Proportion of participants returning to work in the medium term (6 months–1 year)	Event data (return-to-work rates, disability pension rates) or time-to-event data (time span between reporting sick and resumption of work, number of days on sick leave during the follow-up period)	7 (316)	No effect	VERY LOW
Hegewald, 2019 [24]	Interventions to support return to work for people with CHD	39 (8857)	People with CHD	Inpatients, outpatients	Physical conditioning interventions	UC	Proportion of participants returning to work in the medium term (6 months–1 year)	Event data (return-to-work rates, disability pension rates) or time-to-event data (time span between reporting sick and resumption of work, number of days on sick leave during the follow-up period)	5 (510)	No effect	LOW
Hegewald, 2019 [24]	Interventions to support return to work for people with CHD	39 (8857)	People with CHD	Inpatients, outpatients	Combined interventions	UC	Proportion of participants returning to work in the medium term (6 months–1 year)	Event data (return-to-work rates, disability pension rates) or time-to-event data (time span between reporting sick and resumption of work, number of days on sick leave during the follow-up period)	10 (992)	No effect	LOW
Hegewald, 2019 [24]	Interventions to support return to work for people with CHD	39 (8857)	People with CHD	Inpatients, outpatients	Psychological interventions (including health education)	UC	Proportion of participants at work in the long term (>1 to <5 years)	Event data (return-to-work rates, disability pension rates) or time-to-event data (time span between reporting sick and resumption of work, number of days on sick leave during the follow-up period)	3 (239)	No effect	LOW
Hegewald, 2019 [24]	Interventions to support return to work for people with CHD	39 (8857)	People with CHD	Inpatients, outpatients	Physical conditioning interventions	UC	Proportion of participants at work in the long term (>1 to <5 years)	Event data (return-to-work rates, disability pension rates) or time-to-event data (time span between reporting sick and resumption of work, number of days on sick leave during the follow-up period)	2 (156)	No effect	LOW
Hegewald, 2019 [24]	Interventions to support return to work for people with CHD	39 (8857)	People with CHD	Inpatients, outpatients	Combined interventions	UC	Proportion of participants at work in the long term (>1 to <5 years)	Event data (return-to-work rates, disability pension rates) or time-to-event data (time span between reporting sick and resumption of work, number of days on sick leave during the follow-up period)	6 (491)	No effect	VERY LOW
Hegewald, 2019 [24]	Interventions to support return to work for people with CHD	39 (8857)	People with CHD	Inpatients, outpatients	Physical conditioning interventions	UC	Proportion of participants at work in the extended long term (≥5 years)	Event data (return-to-work rates, disability pension rates) or time-to-event data (time span between reporting sick and resumption of work, number of days on sick leave during the follow-up period)	1 (119)	No effect	LOW
Hegewald, 2019 [24]	Interventions to support return to work for people with CHD	39 (8857)	People with CHD	Inpatients, outpatients	Physical conditioning interventions	UC	Proportion of participants at work in the extended long term (≥5 years)	Event data (return-to-work rates, disability pension rates) or time-to-event data (time span between reporting sick and resumption of work, number of days on sick leave during the follow-up period)	4 (350)	No effect	VERY LOW
Hegewald, 2019 [24]	Interventions to support return to work for people with CHD	39 (8857)	People with CHD	Inpatients, outpatients	Psychological interventions (including health education)	UC	Days until return to work	Event data (return-to-work rates, disability pension rates) or time-to-event data (time span between reporting sick and resumption of work, number of days on sick leave during the follow-up period)	2 (125)	No effect	VERY LOW
Hegewald, 2019 [24]	Interventions to support return to work for people with CHD	39 (8857)	People with CHD	Inpatients, outpatients	Physical conditioning interventions	UC	Days until return to work	Event data (return-to-work rates, disability pension rates) or time-to-event data (time span between reporting sick and resumption of work, number of days on sick leave during the follow-up period)	4 (430)	No effect	LOW
Hegewald, 2019 [24]	Interventions to support return to work for people with CHD	39 (8857)	People with CHD	Inpatients, outpatients	Combined interventions	UC	Days until return to work	Event data (return-to-work rates, disability pension rates) or time-to-event data (time span between reporting sick and resumption of work, number of days on sick leave during the follow-up period)	2 (181)	Favour intervention	MODERATE
Hegewald, 2019 [24]	Interventions to support return to work for people with CHD	39 (8857)	People with CHD	Inpatients, outpatients	Work-directed counselling	UC	Days until return to work	Event data (return-to-work rates, disability pension rates) or time-to-event data (time span between reporting sick and resumption of work, number of days on sick leave during the follow-up period)	4 (618)	No effect	LOW
Hegewald, 2019 [24]	Interventions to support return to work for people with CHD	39 (8857)	People with CHD	Inpatients, outpatients	Physical conditioning interventions	UC	Adverse effects: cardiac deaths	NR	2 (285)	No effect	MODERATE
Hegewald, 2019 [24]	Interventions to support return to work for people with CHD	39 (8857)	People with CHD	Inpatients, outpatients	Work-directed counselling	UC	Adverse effects: cardiac deaths	NR	2 (388)	No effect	MODERATE
Hegewald, 2019 [24]	Interventions to support return to work for people with CHD	39 (8857)	People with CHD	Inpatients, outpatients	Combined interventions	UC	Health-related quality of life	Angina Pectoris Quality of Life Questionnaire	1 (87)	No effect	LOW
Hegewald, 2019 [24]	Interventions to support return to work for people with CHD	39 (8857)	People with CHD	Inpatients, outpatients	Combined interventions	UC	Adverse effects: reinfarction	NR	3 (265)	No effect	MODERATE
Herkner, 2007 [30]	Bed rest for acute uncomplicated myocardial infarction	15 (1487)	Patients with acute uncomplicated myocardial infarction	Any settings	Short bed rest	Long bed rest	Total death	Total number	Not measured	NR	Not estimable
Herkner, 2007 [30]	Bed rest for acute uncomplicated myocardial infarction	15 (1487)	Patients with acute uncomplicated myocardial infarction	Any settings	Short bed rest	Long bed rest	Cause-specific death (due to CHD)	NR	Not measured	NR	Not estimable
Herkner, 2007 [30]	Bed rest for acute uncomplicated myocardial infarction	15 (1487)	Patients with acute uncomplicated myocardial infarction	Any settings	Short bed rest	Long bed rest	Reinfarction	NR	Not measured	NR	Not estimable
Kisely, 2015 [31]	Psychological interventions for symptomatic management of non-specific chest pain in patients with normal coronary anatomy	17 (1006)	People presenting with chest pain who have normal anatomy as assessed on clinical history	Inpatients, outpatients	Psychological intervention	No such therapy	Pain intensity	Categorical scales or VAS	Not measured	NR	Not estimable
Kisely, 2015 [31]	Psychological interventions for symptomatic management of non-specific chest pain in patients with normal coronary anatomy	17 (1006)	People presenting with chest pain who have normal anatomy as assessed on clinical history	Inpatients, outpatients	Psychological intervention	No such therapy	Pain diaries	Mean difference in pain scores or recorded frequency of exacerbation of pain	Not measured	NR	Not estimable
Kwong, 2015 [32]	Yoga for secondary prevention of CHD	0 (0)	Patients with CHD	Any settings	Any type of yoga	No intervention or an intervention other than yoga	Mortality	Nr of deaths	Not measured	NR	Not estimable
Kwong, 2015 [32]	Yoga for secondary prevention of CHD	0 (0)	Patients with CHD	Any settings	Any type of yoga	No intervention or an intervention other than yoga	Cardiovascular mortality	Nr of deaths	Not measured	NR	Not estimable
Kwong, 2015 [32]	Yoga for secondary prevention of CHD	0 (0)	Patients with CHD	Any settings	Any type of yoga	No intervention or an intervention other than yoga	Composite cardiovascular events (cardiovascular death, non-fatal myocardial infarction, unstable angina pectoris, resuscitated cardiac arrest, stroke, and cardiac revascularisation procedures)	NR	Not measured	NR	Not estimable
Kwong, 2015 [32]	Yoga for secondary prevention of CHD	0 (0)	Patients with CHD	Any settings	Any type of yoga	No intervention or an intervention other than yoga	Cardiovascular-related hospital admissions	NR	Not measured	NR	Not estimable
Kwong, 2015 [32]	Yoga for secondary prevention of CHD	0 (0)	Patients with CHD	Any settings	Any type of yoga	No intervention or an intervention other than yoga	Adverse effects	NR	Not measured	NR	Not estimable
Long, 2018 [25]	Exercise-based CR for adults with stable angina	7 (581)	Adults with stable angina	Hospital, outpatient clinic, community or home-based environment	exercise-based CR	UC (standard medical care but without any structured training or advice on structured exercise training)	All-cause mortality	NR	3 (195)	No	VERY LOW
Long, 2018 [25]	Exercise-based CR for adults with stable angina	7 (581)	Adults with stable angina	Hospital, outpatient clinic, community or home-based environment	exercise-based CR	UC (standard medical care but without any structured training or advice on structured exercise training)	Acute myocardial infarction (AMI)	NR	3 (254)	No effect	VERY LOW
Long, 2018 [25]	Exercise-based CR for adults with stable angina	7 (581)	Adults with stable angina	Hospital, outpatient clinic, community or home-based environment	exercise-based CR	UC (standard medical care but without any structured training or advice on structured exercise training)	Exercise capacity	VO_2_ max and duration of exercise	5 (267)	Favours intervention	LOW
Long, 2018 [25]	Exercise-based CR for adults with stable angina	7 (581)	Adults with stable angina	Hospital, outpatient clinic, community or home-based environment	exercise-based CR	UC (standard medical care but without any structured training or advice on structured exercise training)	Cardiovascular-related hospital admissions	Combined clinical endpoint (cardiac death, stroke, CABG, PCI, AMI, worsening angina with objective evidence resulting in hospitalisation	1 (101)	No effect	VERY LOW
Long, 2018 [25]	Exercise-based CR for adults with stable angina	7 (581)	Adults with stable angina	Hospital, outpatient clinic, community or home-based environment	exercise-based CR	UC (standard medical care but without any structured training or advice on structured exercise training)	Health-related quality of life	Seattle Angina Quetionnaire and The MacNew Questionnaire	1 (94)	Not pooled	VERY LOW
Long, 2018 [25]	Exercise-based CR for adults with stable angina	7 (581)	Adults with stable angina	Hospital, outpatient clinic, community or home-based environment	exercise-based CR	UC (standard medical care but without any structured training or advice on structured exercise training)	Return to work	NR	NR	Not estimable	NA
Long, 2018 [25]	Exercise-based CR for adults with stable angina	7 (581)	Adults with stable angina	Hospital, outpatient clinic, community or home-based environment	exercise-based CR	UC (standard medical care but without any structured training or advice on structured exercise training)	Adverse events	NR	1 (101)	Not pooled	VERY LOW
McDonagh, 2023 [26]	Home-based versus centre-based CR	24 (3046)	Patients with heart disease	Mixed (Rehabilitation centre, home-based)	Home-based CR	Centre-based CR	Total mortality	Number of deaths	12 (1647)	No effect	LOW
McDonagh, 2023 [26]	Home-based versus centre-based CR	24 (3046)	Patients with heart disease	Mixed (Rehabilitation centre, home-based)	Home-based CR	Centre-based CR	Exercise capacity ≤ 12 months	VO_2_ peak, 6 min walk test	24 (2343)	No effect	LOW
Mc Donagh, 2023 [26]	Home-based versus centre-based CR	24 (3046)	Patients with heart disease	Mixed (Rehabilitation centre, home-based)	Home-based CR	Centre-based CR	Exercise capacity > 12 months	VO_2_ peak, 6 min walk test	3 (1074)	No effect	MODERATE
Mc Donagh, 2023 [26]	Home-based versus centre-based CR	24 (3046)	Patients with heart disease	Mixed (Rehabilitation centre, home-based)	Home-based CR	Centre-based CR	Withdrawal from the exercise programme	Number of completers	23 (2638)	No effect	LOW
Mc Donagh, [26] 2023	Home-based versus centre-based CR	24 (3046)	Patients with heart disease	Mixed (Rehabilitation centre, home-based)	Home-based CR	Centre-based CR	HRQoL	Short-Form Health Survey (SF-36), Sickness Impact Profile, Nottingham Health Profile	18 (2207)	Not pooled	MODERATE
Richards, 2017 [27]	Psychological interventions for CHD	35 (10,703)	People with CHD	Centre- or home-based (with/without telephone support)	Psychological intervention with/without other rehabilitation	UC or other rehabilitation intervention	Total mortality	Nr. of deaths	23 (7776)	No effect	MODERATE
Richards, 2017 [27]	Psychological interventions for CHD	35 (10,703)	People with CHD	Centre- or home-based (with/without telephone support)	Psychological intervention with/without other rehabilitation	UC or other rehabilitation intervention	Cardiac mortality	Nr. of deaths	11 (4792)	Favour intervention	LOW
Richards, 2017 [27]	Psychological interventions for CHD	35 (10,703)	People with CHD	Centre- or home-based (with/without telephone support)	Psychological intervention with/without other rehabilitation	UC or other rehabilitation intervention	Non-fatal MI	NR	13 (7845)	No effect	LOW
Richards, 2017 [27]	Psychological interventions for CHD	35 (10,703)	People with CHD	Centre- or home-based (with/without telephone support)	Psychological intervention with/without other rehabilitation	UC or other rehabilitation intervention	Revascularisation (CABG and PCI combined)	NR	13 (6822)	No effect	MODERATE
Richards, 2017 [27]	Psychological interventions for CHD	35 (10,703)	People with CHD	Centre- or home-based (with/without telephone support)	Psychological intervention with/without other rehabilitation	UC or other rehabilitation intervention	Anxiety	NR	12 (3165)	Favour intervention	LOW
Richards, 2017 [27]	Psychological interventions for CHD	35 (10,703)	People with CHD	Centre- or home-based (with/without telephone support)	Psychological intervention with/without other rehabilitation	UC or other rehabilitation intervention	Depression	NR	19 (5829)	Favour intervention	LOW
Richards, 2017 [27]	Psychological interventions for CHD	35 (10,703)	People with CHD	Centre- or home-based (with/without telephone support)	Psychological intervention with/without other rehabilitation	UC or other rehabilitation intervention	Stress	NR	8 (1255)	Favour intervention	VERY LOW
Tully, 2021 [28]	Psychological and pharmacological interventions for depression in patients with CAD	37	Patients with CAD	Outpatient, inpatient	Psychological treatment	UC	Depression symptoms—short term (end of treatment)	Objective and self-reported measures of depression symptoms	10 (1226)	Favour intervention	LOW
Tully, 2021 [28]	Psychological and pharmacological interventions for depression in patients with CAD	37	Patients with CAD	Outpatient, inpatient	Psychological treatment	UC	Depression remission—short term (end of treatment)	Objective and self-report measures of depression	3 (862)	No effect	LOW
Tully, 2021 [28]	Psychological and pharmacological interventions for depression in patients with CAD	37	Patients with CAD	Outpatient, inpatient	Psychological treatment	UC	All-cause mortality—short term (end of treatment)	Mortality records	2 (324)	No effect	VERY LOW
Tully, 2021 [28]	Psychological and pharmacological interventions for depression in patients with CAD	37	Patients with CAD	Outpatient, inpatient	Psychological treatment	UC	Cardiovascular mortality—long term (≥6 months after the end of treatment)	Cause of death according to standardised criteria on mortality records	2 (2720)	No effect	Not estimable
Tully, 2021 [28]	Psychological and pharmacological interventions for depression in patients with CAD	37	Patients with CAD	Outpatient, inpatient	Psychological treatment	UC	Myocardial infarction—short term	NR	NR	Not estimable	NA
Tully, 2021 [28]	Psychological and pharmacological interventions for depression in patients with CAD	37	Patients with CAD	Outpatient, inpatient	Psychological treatment	Psychological treatment/clinical management	Depression symptoms—short term (at the end of treatment)	Objective and self-reported measures of depression symptoms	3 (219)	NR	NA
Tully, 2021 [28]	Psychological and pharmacological interventions for depression in patients with CAD	37	Patients with CAD	Outpatient, inpatient	Psychological treatment	Psychological treatment/clinical management	Depression remission—short term (at the end of treatment)	Hamilton Rating Scale for Depression	1 (83)	No effect	LOW
Tully, 2021 [28]	Psychological and pharmacological interventions for depression in patients with CAD	37	Patients with CAD	Outpatient, inpatient	Psychological treatment	Psychological treatment/clinical management	All-cause mortality—short term	NR	NR	Not estimable	NA
Tully, 2021 [28]	Psychological and pharmacological interventions for depression in patients with CAD	37	Patients with CAD	Outpatient, inpatient	Psychological treatment	Psychological treatment/clinical management	Cardiovascular mortality—short term	NR	NR	Not estimable	NA
Tully, 2021 [28]	Psychological and pharmacological interventions for depression in patients with CAD	37	Patients with CAD	Outpatient, inpatient	Psychological treatment	Psychological treatment/clinical management	Myocardial infarction—short term	NR	NR	Not estimable	NA
Tully, 2021 [28]	Psychological and pharmacological interventions for depression in patients with CAD	37	Patients with CAD	Outpatient, inpatient	Pharmacological (all antidepressant medications and drug therapies used explicitly for treating depressive disorders)	Placebo	Depression symptoms—short term (end of treatment)	Objective and self-reported measures of depression symptoms	8 (750)	Favour intervention	LOW
Tully, 2021 [28]	Psychological and pharmacological interventions for depression in patients with CAD	37	Patients with CAD	Outpatient, inpatient	Pharmacological (all antidepressant medications and other drug therapies used explicitly for treating depressive disorders)	Placebo	Depression remission—short term (end of treatment)	Hamilton Rating Scale for Depression	4 (646)	Favour intervention	MODERATE
Tully, 2021 [28]	Psychological and pharmacological interventions for depression in patients with CAD	37	Patients with CAD	Outpatient, inpatient	Pharmacological (all antidepressant medications and other drug therapies used explicitly for treating depressive disorders)	Placebo	All-cause mortality—short term (end of treatment)	Mortality records	2 (437)	No effect	VERY LOW
Tully, 2021 [28]	Psychological and pharmacological interventions for depression in patients with CAD	37	Patients with CAD	Outpatient, inpatient	Pharmacological (all antidepressant medications and other drug therapies used explicitly for treating depressive disorders)	Placebo	Cardiovascular mortality—short term	NR	NR	Not estimable	NA
Tully, 2021 [28]	Psychological and pharmacological interventions for depression in patients with CAD	37	Patients with CAD	Outpatient, inpatient	Pharmacological (all antidepressant medications and other drug therapies used explicitly for treating depressive disorders)	Placebo	Myocardial infarction—short term	Standardised criteria for fatal or non-fatal myocardial infarction	3 (728)	No effect	VERY LOW
Tully, 2021 [28]	Psychological and pharmacological interventions for depression in patients with CAD	37	Patients with CAD	Outpatient, inpatient	Pharmacological intervention 1	Pharmacological intervention 2	Depression symptoms—short term	Hamilton Rating Scale for Depression	4 (442)	Not pooled	NA
Tully, 2021 [28]	Psychological and pharmacological interventions for depression in patients with CAD	37	Patients with CAD	Outpatient, inpatient	Pharmacological intervention 1	Pharmacological intervention 2	Depression remission—short term	Objective and self-reported measures of depression symptoms	3 (243)	Not pooled	NA
Tully, 2021 [28]	Psychological and pharmacological interventions for depression in patients with CAD	37	Patients with CAD	Outpatient, inpatient	Pharmacological intervention 1	Pharmacological intervention 2	All-cause mortality	Mortality records	1 (149)	No effect	VERY LOW
Tully, 2021 [28]	Psychological and pharmacological interventions for depression in patients with CAD	37	Patients with CAD	Outpatient, inpatient	Pharmacological intervention 1	Pharmacological intervention 2	Cardiovascular mortality—short term	NR	NR	Not estimable	NA
Tully, 2021 [28]	Psychological and pharmacological interventions for depression in patients with CAD	37	Patients with CAD	Outpatient, inpatient	Pharmacological intervention 1	Pharmacological intervention 2	Myocardial infarction—short term	Standardised criteria for fatal or non-fatal myocardial infarction	3 (396)	Not pooled	NA

Legend: coronary artery disease: CAD; coronary heart disease: CHD; cardiac rehabilitation: CR; Coronary Artery Bypass Grafting: CABG; myocardial infarction: MI; Not Available: NA; Number: Nr; Not Reported: NR; Numeric Rating Scale: NRS; percutaneous coronary intervention: PCI; Spielberger State-Trait Anxiety Inventory: STAI; Usual care: UC; Visual Analogue Scale: VAS.

## Data Availability

No new data were generated or analysed in support of this research.

## Supplementary Materials

The following supporting information can be downloaded at https://www.mdpi.com/article/10.3390/jcm13133662/s1: Table S1: AMSTAR 2 Quality Assessment of Cochrane Systematic Reviews.
